# Soil organic carbon cycling in response to simulated soil moisture variation under field conditions

**DOI:** 10.1038/s41598-021-90359-4

**Published:** 2021-05-25

**Authors:** Shikha Singh, Melanie A. Mayes, Avat Shekoofa, Stephanie N. Kivlin, Sangeeta Bansal, Sindhu Jagadamma

**Affiliations:** 1 Department of Biosystems Engineering and Soil Science, University of Tennessee, 2506 E.J Chapman Drive, Knoxville, TN 37996 USA; 2 Environmental Sciences Division & Climate Change Science Institute, Oak Ridge National Laboratory, Oak Ridge, TN USA; 3 Department of Plant Sciences, University of Tennessee, Knoxville, TN USA; 4 Department of Ecology and Evolutionary Biology, University of Tennessee, Knoxville, TN USA

**Keywords:** Biogeochemistry, Carbon cycle

## Abstract

The combination of extended dry periods and high intensity rainfall, common in the southeastern US, leads to greater variability in soil moisture and consequently increases uncertainty to microbial processes pertinent to soil carbon (C) mineralization. However, field-based findings on soil moisture sensitivity to soil C cycling are very limited. Therefore, a field experiment was conducted in 2018 and 2019 on a soybean (*Glycine max* L.) cropland in the southeastern US with three soil moisture treatments: drought (simulated using rainout-shelter from June to October in each year), rainfed (natural precipitation), and irrigated (irrigation and precipitation). Soil respiration was measured weekly from May to November in both years. Soil samples were collected multiple times each year from 0–5, 5–15, and 15–30 cm depths to determine microbial biomass C (MBC), extractable organic C (EOC), hydrolytic enzyme activities, and fungal abundance. The cumulative respiration under drought compared to other treatments was lower by 32% to 33% in 2018 and 38% to 45% in 2019. Increased MBC, EOC, and fungal abundance were observed under drought than other treatments. Specific enzyme activity indicated fewer metabolically active microbes under drought treatment compared to rainfed and irrigated treatments. Also, maintenance of enzyme pool was observed under drought condition. These results provide critical insights on microbial metabolism in response to soil moisture variation and how that influences different pools of soil C under field conditions.

## Introduction

Elevated atmospheric concentrations of CO_2_ and other greenhouse gases (GHGs) are expected to increase earth’s surface temperatures and alter precipitation regimes^1,2^. A global model intercomparison study revealed that future global water cycle will be altered by climatic changes, leading to changes in amount, frequency, timing, and intensity of precipitation patterns, and frequency and duration of drought^3–5^. Historical climate data suggest that rainfall patterns have already shifted over the past centuries, and there has been a 10% increase in total precipitation in the United States^6,7^ with approximately half of the increase being due to very high intensity rainfall events of > 40 mm day^−1^. Increased drought periods interspersed with high intensity precipitation events will lead to greater variability in soil moisture regimes^8^ with potential influence on sources and sinks of GHGs in terrestrial ecosystems^9^. Although terrestrial ecosystems are highly sensitive to changes in precipitation patterns^10^, the extent to which the predicted altered rainfall regimes influence soil organic carbon (SOC) cycling and storage in terrestrial ecosystems is highly variable.

Soil moisture content acts as a key variable influencing microbial processing of SOC^11,12^. Extremes of moisture conditions (e.g., drought or flooding) tend to adversely affect the rate of microbial respiration. Drought limits the enzyme and substrate diffusion by breaking water films and flooded conditions promote less-efficient anaerobic decomposition by limiting oxygen diffusion^13^. The response of microbial respiration to changing precipitation patterns has been well studied^14–17^. However, the impacts of droughts, whose intensity and frequency are predicted to increase under changing climate^18^, on microbial respiration remain less certain. The limited available data based on in situ moisture manipulation experiments revealed variable effects of moisture stress on microbial respiration^19–21^. Soil microbial respiration response to drought may differ between growing and non-growing seasons^22^, resource availability^23^, and/or soil microbial abundance and composition^24^. Intriguingly, historical legacies of prior drought conditions can impart a signature of reduced respiration even after cessation of drought conditions. Yet, it is unclear when and how long prior drought legacies persist across ecosystems^25^. Theoretically, for a drought legacy to persist in soil microbiomes, drought-tolerant microbial physiological acclimation (adjustment within a microbial lifetime), adaptation (genetically-linked selection across microbial generations), and/or shifts in microbial composition to drought-tolerant taxa must occur^26,27^. Regardless, some studies observed legacy effects to soil moisture changes^28,29^ while some have not^25^. The inconsistencies in these responses could be attributed to functional diversity of microbial communities in soils or environmental factors but were inconclusive due to a smaller number of studies conducted^26^.

Predicting microbial functional responses to soil water variability is challenging. Water deficit or drought conditions between rainfall events can result in reduced microbial respiration as a result of microbial stress^30–33^. Under such conditions, microbes tend to acclimate/adapt through several mechanisms such as accumulating osmolytes that help moderate osmotic stresses^34^, undergoing dormancy by investing energy in creating structures around microbial cells such as endospores that support long-term survival^35^, producing extracellular polymers that act like sponges for microbial cells to delay desiccation^33^ or changing microbial community composition by the selection of drought tolerant taxa^36,37^. Conversely, precipitation events can increase microbial activity as substrate diffusion is enhanced, leading to increased CO_2_ fluxes, although this response is not consistent^38^. Intense or longer precipitation events can, however, decrease microbial respiration due to decreased oxygen availability^39^. Also, sudden increases in soil moisture could cause microbial cell lysis leading to a decrease in total microbial population; however, cell lysis can readily supply substrates to the surviving microbes, so the net effect on microbial respiration is less certain. As a result of the tight coupling of microbial functioning to soil moisture described above, it is difficult to anticipate a priori the responses of microbial respiration to soil moisture changes. Most past studies were conducted by manipulating soil moisture in laboratory microcosms, where environmental conditions were controlled, soil structures were disturbed, and continuous C inputs were prevented. These laboratory-scale experiments are valuable, but they miss the opportunity to study soil biogeochemical changes to moisture variability in tandem with other biotic and abiotic changes. Field-based experiments offer a better representation of native ecological conditions compared to laboratory experiments, but field studies on soil moisture-microbial activity relationship are sparse.

Thus, the present study was conducted with the objectives to: (1) assess the dependence of SOC mineralization to in situ soil moisture changes, and (2) determine if in situ moisture manipulation has legacy effects on soil microbial activity. The hypotheses were: (1) soil moisture stress would negatively affect microbial respiration; (2) soil microbes would acclimate to moisture stress, so microbial activity would continue during drought, despite at reduced rate; and (3) legacy effect on microbial activity would be observed after cessation of moisture stress.

## Materials and methods

### Study description and experimental design

The study site was located at the West Tennessee Research and Education Center of the University of Tennessee in Jackson, TN (35°37′22″ N, 88°50′47″ W; elevation 125 m). The study site experiences a mean annual temperature and precipitation of 15.6 °C and 1375 mm, respectively (based on 30-year average). Soils of the study site are classified as well-drained Lexington series (fine-silty, mixed, thermic, Ultic Hapludalfs) with 0 to 2% slope. The soil moisture manipulation was conducted on a no-till soybean (*Glycine max* L.) cropping system in 2018 and 2019. All guidelines were adhered to in the production of this study. We obtained permissions to grow and collect soybean (*Glycine max* L.). Soil organic carbon content at the study site was 14 and 12 g kg^−1^ in 2018 and 2019, respectively. The treatments were arranged in a completely randomized design (CRD) with three replicates and each plot was 3.4 m × 3 m with a 1 m alley between the plots. The soybeans (genotype Ellis) were planted in first week of May in both years with four rows per plot and fertilized with phosphorus, potassium, and sulfur at the rate of 33.6 kg ha^−1^, 112 kg ha^−1^, and 17 kg ha^−1^, respectively in 2018 and 33.6 kg ha^−1^, 90 kg ha^−1^, and 13.5 kg ha^−1^, respectively, in 2019 based on soil test results. All plots were treated with 3.36 kg ha^−1^ alachlor (2-Chloro-N-(2,6-diethylphenyl)-N-(methoxymethyl)acetamide) and 0.42 kg ha^−1^ metribuzin (4-Amino-6-tert-butyl-3-methylsulfanyl-1,2,4-triazin-5-one) for pre-emergence weed suppression and 0.13 kg ha^−1^ clethodim (2-[1-[[(E)-3-chloroprop-2-enoxy] amino] propylidene]-5-(2-ethylsulfanylpropyl) cyclohexane-1,3-dione) for post-emergence weed control^40^.

**Figure 1:**
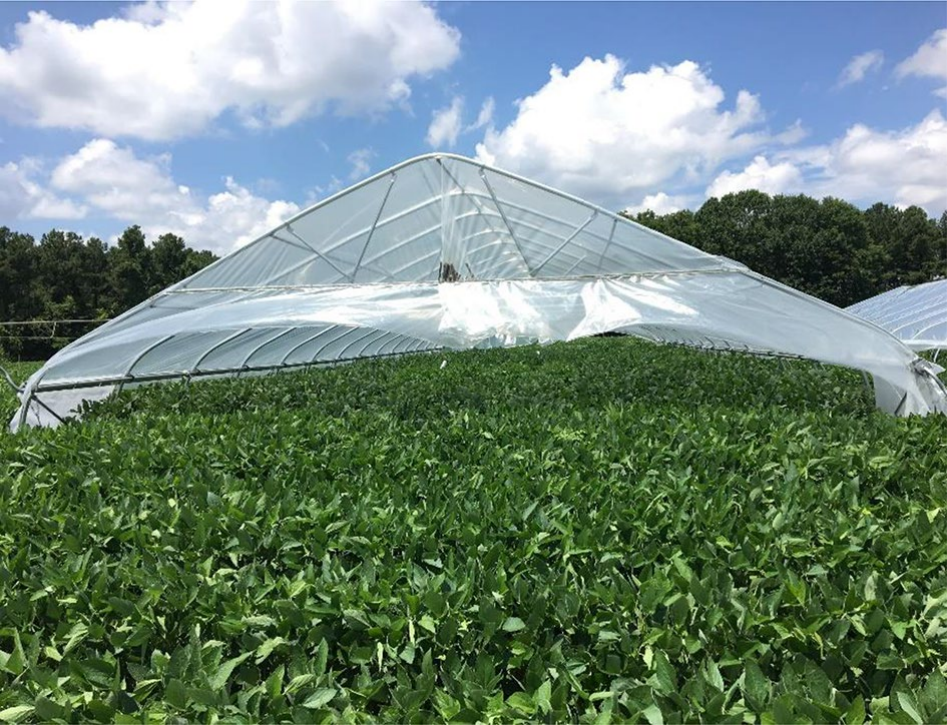
The rainout shelters placed to simulate drought. The picture was taken by Dr. Avat Shekoofa.

There were three soil moisture treatments, viz., (i) drought, (ii) rainfed, and (iii) irrigated. Drought treatment was imposed on the experimental plots by covering the plots using portable rainout shelters (Fig. 1). The shelters were established in the plots from second week of June to first week of October each year. The crops under shelter (drought treatment) as well under rainfed and irrigated treatments received 16 cm and 16.5 cm of water from rainfall in May (2018 and 2019) for seedling emergence before the shelters were established for moisture manipulation. The rainfed treatment received rainfall throughout the entire growing season in both years and the irrigated treatment received 11 cm of water in 2018 and 8 cm in 2019 through irrigation, in addition to the rainfall, at critical growth stages from flowering to seed fill. The total amount of water received when shelters were in place (mid-June to first week of October) in 2018 and 2019 were 87 cm and 67 cm, respectively for the rainfall treatment, and 98 and 75 cm, respectively, for the irrigated treatment.

### Soil sampling and analyses

Soil samples were collected from 0–5, 5–15, and 15–30 cm depths in May, July, September, and November of 2018 and 2019. Multiple soil cores were collected from the inter-row area of the middle two rows of plants in each plot using a probe of 2.5 cm diameter and then composited to obtain a single sample per plot. Soil sampling in May was conducted before moisture manipulation to determine the baseline soil properties, in July and September when rain exclusion shelters were in place to determine the effect of moisture stress, and in November after the shelters were removed to determine the short-term legacy effect of moisture manipulations on microbial activity. The samples were kept in airtight plastic bags and transferred to the laboratory in coolers with ice packs. Gravimetric soil moisture content was determined immediately followed by sieving the fresh samples through 2 mm sieve to remove plant materials and rocks.

Soil microbial biomass C (MBC) was determined using the chloroform fumigation and extraction method^41^. Briefly, 10 g of fresh soil sample was fumigated in the dark for 48 h. Fumigated samples were extracted using 0.5 M K_2_SO_4_ (1:4 soil:0.5 M K_2_SO_4_), shaken at 200 revolutions per minute for 1 h, and centrifuged at 3500 rpm for 3 min. The supernatant was passed through a 0.45 μm filter paper. Similarly, 10 g of unfumigated samples were also extracted. Filtrates from both fumigated and unfumigated samples were analyzed for extractable C using a CN analyzer (Vario TOC cube in liquid mode, Elementar, Hanau, Germany). Soil MBC was determined as the difference in C concentration between fumigated and unfumigated soil samples and dividing that by the k_EC_ (extraction efficiency) value of 0.45^42^. The C concentration of unfumigated soil was considered as extractable organic C (EOC)^43^.

Soil fungal abundance was measured after extracting the fungal hyphae from the soils by following the protocol described in^44^. Briefly, soil was dispersed using 3% sodium hexametaphosphate solution followed by filtering a 5 mL aliquot through 0.45 μm filter paper to separate the fungal hyphae from liquid. The hyphae trapped on the filter paper were then stained using 0.01% acid fuchsin dye and mounted on a slide. This was followed by adding polyvinyl alcohol-lactic acid-glycerol (PVLG) mounting media on the slides and carefully placing a cover slip. Both septate (i.e., decomposer and pathogenic fungi) and aseptate hyphae (i.e., arbuscular mycorrhizal fungi) were quantified using a compound microscope under 20× magnification with 50 fields of view using the grid-line intercept method^45^. Hyphal lengths were reported as sum of both septate and aseptate hyphae.

The activity of six extracellular hydrolytic enzymes, viz., α-glucosidase (AG), β-glucosidase (BG), cellobiohydrolase (CBH), β-xylosidase (XYL), N-acetyl glucosaminidase (NAG), and phosphatase (AP) were determined using 96-well plates method outlined by^46^. The reference substrates for AG, BG, CBH, XYL, NAG, and PHOS were 4-methylumbelliferone (MUB)-α-d-glucopyranoside, 4-MUB-β-d-glucopyranoside, 4-MUB-β-d-cellobioside, 4-MUB-B-d-xylopyranoside, 4-MUB-N-acetyl-β-d-glucosaminide, and 4-MUB phosphate, respectively. Briefly, 2.75 g of soil sample was homogenized in a Waring blender with 91 mL of 50 mM sodium acetate buffer on high setting for one minute. Following that, 800 μL of the soil slurry was transferred into each of the 8 wells of one column. Another plate was prepared to create a standard curve for each sample at 25 °C. Each column in the standard curve plate contained a soil slurry with a different concentration of the 4-MUB standard (0, 2.5, 5, 10, 25, 50, and 100 μM) in each of the wells. After adding 12 samples in a plate, 200 μL of 200 μM respective substrates were added. The plates were incubated at 25 °C for 3 h. Post incubation, the plates were centrifuged at 350×*g* for 3 min and 250 μL of the supernatant from each well was transferred to a 96-well black plate. A plate reader (Synergy-BioTek Instruments, Inc., Vermont, USA) was used to measure fluorescence at wavelengths 365 nm and 450 nm for excitation and emission, respectively. The standard curve plates were used to construct a linear standard curve to determine each enzymes’ activity for each sample as nmol g^−1^ dry soil h^−1^. Total extracellular enzyme activity was calculated as the sum of AG, BG, CBH, XYL, NAG, and PHOS. Specific enzyme activity was calculated by dividing the total enzyme activity by MBC^47^.

### Soil surface CO_2_ measurement

Soil surface CO_2_ emissions were measured from June through November in 2018 and 2019. Gas sampling was completed on weekly intervals depending on weather conditions. After the shelters were removed in early October, three gas sampling were conducted on biweekly intervals. Vented polyvinyl chloride static chamber collars (20 cm diameter × 10 cm height) were installed between two middle rows of soybean in each plot in late May and only removed while harvesting soybeans and then re-placed. Gas sampling was typically done between 9:00 a.m. and 12:00 p.m. to minimize the effect of temperature on CO_2_ fluxes. Chamber air temperature, soil temperature, and soil moisture were measured during each sampling time at each plot. After securely placing a lid on the chamber collars, gas samples were collected at 0, 20, and 40 min using a 15 mL syringe and then transferred to 12 mL evacuated exetainer vials (Labco, United Kingdom). Gas samples were analyzed for CO_2_ concentrations using a flame ionization detector on a gas chromatograph (Shimadzu GC-2014, Japan) using N_2_ as a carrier gas. Soil CO_2_ fluxes (F) were calculated as the change in the headspace CO_2_ concentration over time within the enclosed chamber volume^48,49^ as shown in Eq. (1).

1 $$F = \left( {g/t} \right) \, \left( {V/A} \right) \, K$$

where (g/t) is the linear change in CO_2_ concentrations inside the chamber, V is the volume of the chamber, A is the surface area circumscribed by the chamber, and K is the time conversion coefficient. Cumulative fluxes for each treatment from June through November were calculated using a linear interpolation method.

### Statistical analyses

Statistical analyses for each year were conducted using the GLIMMIX procedure in SAS v9.4^50^. Analysis of variance (ANOVA) was conducted for each soil depth and each sampling time separately to compare the impacts of in situ moisture manipulations on CO_2_ fluxes, MBC, EOC, extracellular enzyme activity, and specific enzyme activity with moisture treatments as fixed effects and replication as random effects. The effect of time and moisture treatments and their potential interactions on the above-mentioned parameters were determined using a repeated measures ANOVA with sampling time as the repeated measure variable. Mean differences were considered significant at *p* ≤ 0.05. The mean separation was done using Tukey’s test. The results from the repeated measures ANOVA for all the parameters are included in Table 1.

**Table 1:** Repeated measures ANOVA statistics (p value) for soil moisture (treatments) and sampling time effects on various measured parameters.

Soil depth (cm)	Measured parameters	2018			2019		
		Trt	Time	Trt × time	Trt	Time	Trt × time
0–5	CO_2_ emissions	< 0.0001	< 0.0001	0.0004	0.0002	< 0.0001	0.0002
	Extractable organic C	< 0.0001	0.009	NS	< 0.0001	< 0.0001	0.007
	Microbial biomass C	0.086	0.0001	NS	0.004	< 0.0001	0.0002
	Hyphal length	NS	< 0.0001	NS	0.002	0.003	0.006
	Total enzyme activity	< 0.0001	< 0.0001	< 0.0001	< 0.0001	0.0003	0.0001
	Specific enzyme activity	0.0003	< 0.0001	0.001	< 0.0001	0.006	0.044
5–15	Extractable organic C	0.0001	0.038	0.004	0.001	0.0001	0.006
	Microbial biomass C	0.0002	NS	NS	0.001	0.044	0.001
	Hyphal length	NS	NS	NS	0.023	0.0001	0.001
	Total enzyme activity	0.015	< 0.0001	0.001	0.0002	< 0.0001	0.001
	Specific enzyme activity	< 0.0001	< 0.0001	NS	0.001	0.004	0.259
15–30	Extractable organic C	NS	< 0.0001	NS	0.009	0.0002	0.007
	Microbial biomass C	NS	0.001	NS	NS	0.016	0.640
	Total enzyme activity	NS	NS	NS	< 0.0001	NS	NS
	Specific enzyme activity	NS	0.001	0.026	< 0.0001	< 0.0001	0.0002

NS means non-significant.

## Results

### Climatic conditions, soil temperature, and soil water

**Figure 2:**
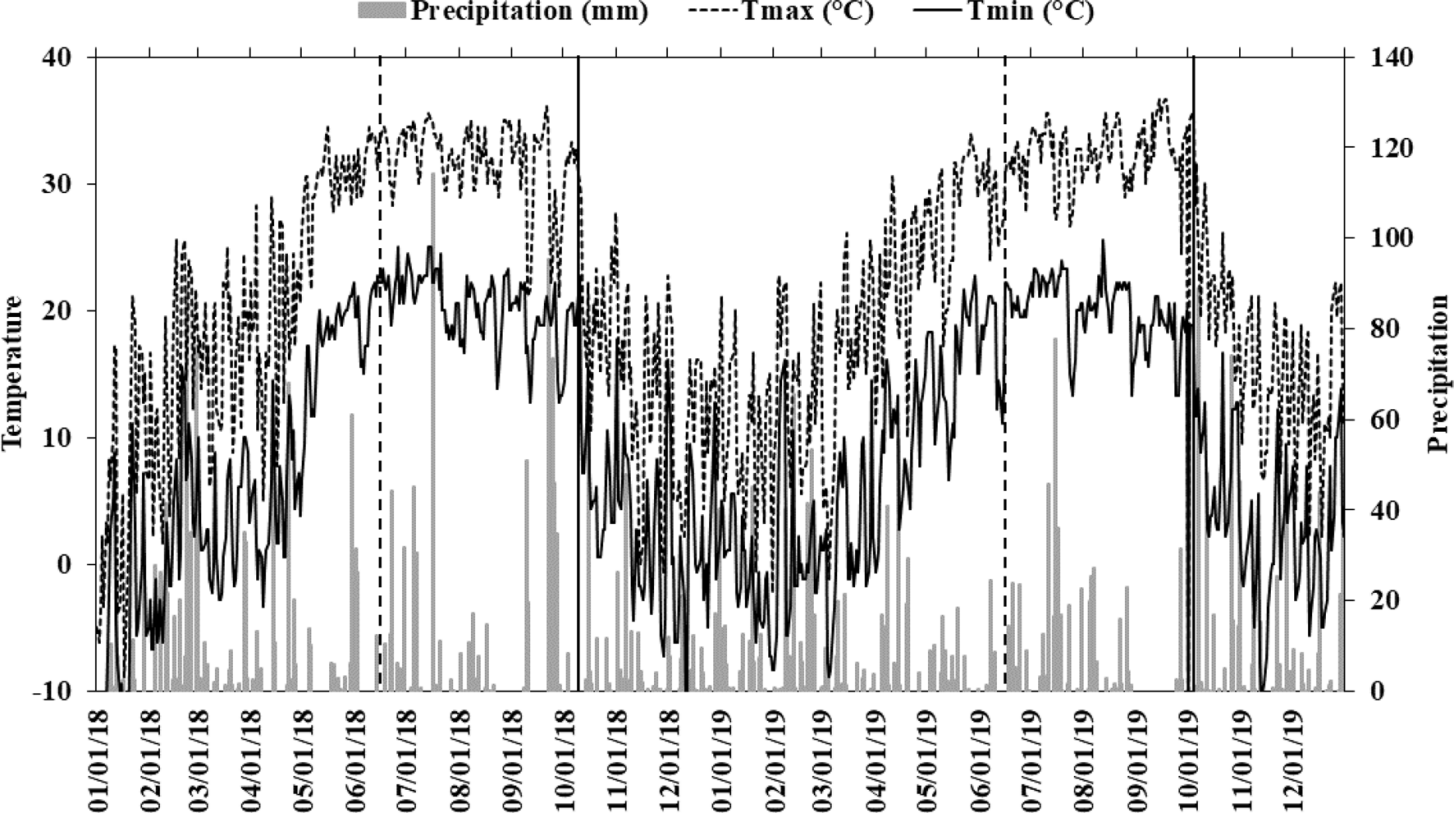
Daily maximum and minimum air temperature and precipitation during 2018 and 2019. Tmax, maximum air temperature; Tmin, minimum air temperature. The vertical dashed lines represents the shelter establishment dates and the vertical solid lines represent the shelter removal dates during 2018 and 2019.

**Figure 3:**
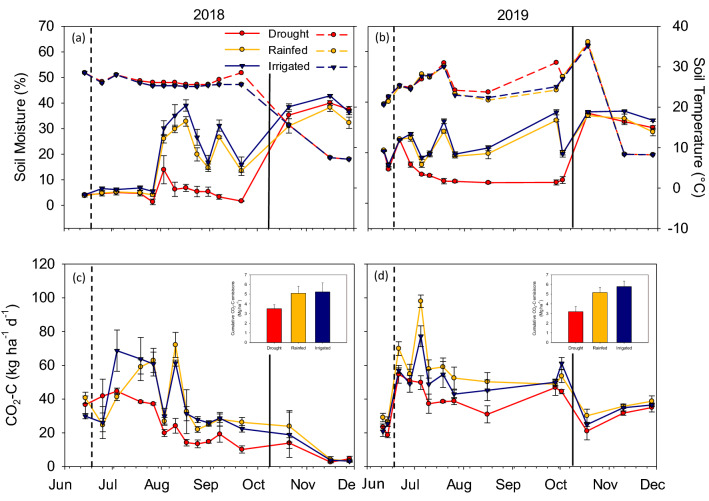
Soil temperature and soil moisture (0–5 cm) as influenced by moisture treatments in 2018 (**a**) and 2019 (**b**). Dashed line in the legend represents the soil temperature and solid line represents the soil moisture. Daily CO_2_ fluxes during the growing season in 2018 (**c**) and 2019 (**d**). Legend for daily CO_2_ fluxes is same as soil moisture. Vertical dashed line represents shelter establishment date and solid line represents shelter removal date. Inset: cumulative CO_2_ emissions during the growing season. Error bars represent standard error (n = 3).

Daily maximum and minimum air temperature, and precipitation for 2018 and 2019 are shown in Fig. 2. The area received 103 and 83 cm of precipitation from May through first week of October in 2018 and 2019, respectively. Mean air temperature during 2018 and 2019 was similar during the growing season. Soil temperature and moisture for 2018 and 2019 are shown in Fig. 3a,b, respectively. In 2018, soil temperature was the same for all three treatments until early July, after that the drought treatment showed 0.5–2 °C higher temperature than the rainfed and irrigated treatments until the shelters were removed. The temperature reduced to 7 °C for all the three treatments by the end of November. In 2019, soil temperatures were similar in the three treatments until the last week of July, and after that the drought treatment showed 0.5–5 °C higher temperature than the rainfed and irrigated treatments. Similar to 2018, soil temperature was reduced to 8 °C by the end of November. Soil moisture was similar in the three treatments until mid-July in 2018 and after that, soil moisture was consistently lowest in drought treatment (Fig. 3a). After removal of shelters in early October, soil moisture increased in the three treatments for both years and no differences were observed among the three treatments. Similar trend in soil moisture was observed in 2019 (Fig. 3b).

### Soil CO_2_ efflux in response to soil moisture

The repeated measures ANOVA showed that treatment, time of sampling, and their interaction strongly influenced CO_2_ emissions at *p* ≤ 0.05 (Table 1). Soil CO_2_ efflux consistently followed soil moisture trends with the lowest CO_2_ efflux observed from the drought treatment compared to rainfed and irrigated treatments in both years (Fig. 3c,d). In 2018, the baseline sample collected before shelter placement on June 14 did not show differences in CO_2_ emissions across the moisture treatments (Fig. 3c). From mid-July 2018, CO_2_ emissions from the drought treatment were lower than rainfed and irrigated treatments until the first week of October (Fig. 3c). After the shelters were removed in the first week of October, no differences in CO_2_ emissions were observed among the three treatments and the fluxes were much lower than previous sampling events. In 2019 also, the baseline data showed no differences among the three treatments and after that the drought treatment exhibited the lowest CO_2_ emissions (Fig. 3d) until early October. Similar to 2018, the last three gas sampling events in 2019 after shelter removal showed reduced emissions with no differences among the three treatments. Cumulative CO_2_ emissions from June through November under drought treatment was 32% and 33% lower than rainfed and irrigated treatments, respectively, in 2018 (see inset in Fig. 3c), and 38% and 45% lower in 2019 (see inset in Fig. 3d).

### Active soil C pools in response to soil moisture

**Figure 4:**
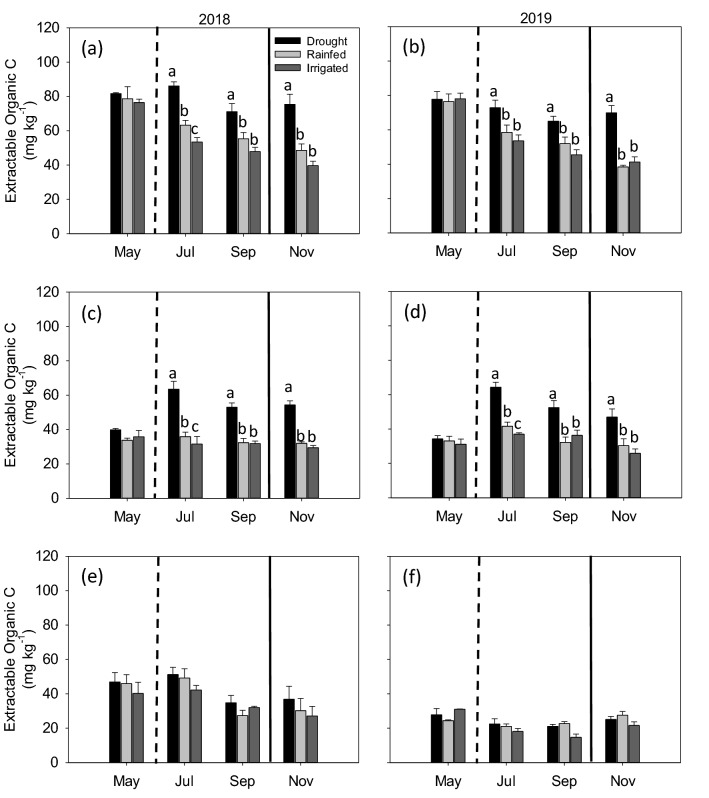
Extractable organic carbon concentrations under drought, rainfed, and irrigated treatments at 0–5 (**a**,**b**), 5–15 (**c**,**d**), and 15–30 (**e**,**f**) cm depths during 2018 and 2019. Different letters represent statistical significance at p ≤ 0.05 at each time point. No letters mean no statistical significance. Error bars represent standard error (n = 3). Sampling in May represents baseline and in November represents post shelter removal. Vertical dashed line represents shelter establishment date and solid line represents shelter removal date.

**Figure 5:**
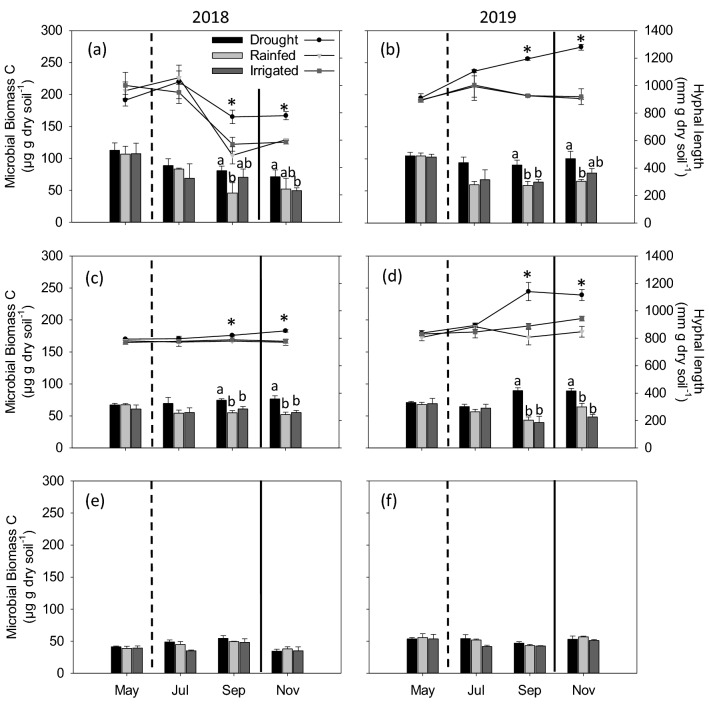
Microbial biomass carbon concentrations (bar graphs) under drought, rainfed, and irrigated treatments at 0–5 (**a**,**b**), 5–15 (**c**,**d**) and 15–30 (**e**,**f**) cm depths during 2018 and 2019. Different letters represent statistical significance at p ≤ 0.05 at each time point. No letters mean no statistical significance. The secondary axis represents the hyphal length (line graph) in each treatment at each time point. The hyphal length was not determined for 15–30 cm depth (**e**,**f**) as microbial biomass carbon did not show significant differences between treatments. Asterisk represents statistical significance at p ≤ 0.05 at each time point. Error bars represent standard error (n = 3). Sampling in May represents baseline and in November represents post shelter removal. Vertical dashed line represents shelter establishment date and solid line represents shelter removal date.

In general, EOC showed differences among moisture treatments at 0–5 (Fig. 4a,b) and 5–15 cm (Fig. 4c,d) depths, not at 15–30 cm (Fig. 4e,f) depth for both years. There were no differences among the three treatments in May 2018 and 2019, before the shelter establishment, for both the 0–5 and 5–15 cm depths. However, in both years, drought treatment showed higher EOC compared to rainfed and irrigated treatments in July and September at 0–5 (Fig. 4a,b) and 5–15 cm (Fig. 4c,d) depths. In November, 6 weeks after the shelters were removed, drought treatment still showed higher EOC than rainfed and irrigated treatments for both 0–5 and 5–15 cm depths. More specifically, at 0–5 cm depth, EOC under drought averaged 55% and 90% higher in November 2018 (Fig. 4a) and 82% and 69% higher in November 2019 (Fig. 4b) than rainfed and irrigated treatments, respectively. The EOC showed a consistent decrease with depth and over time within each depth for all the three treatments.

Similar to EOC, MBC also showed differences among the moisture treatments at 0–5 cm (Fig. 5a,b) and 5–15 cm (Fig. 5c,d) depths for both years while no differences were observed at 15–30 cm depth (Fig. 5e,f). In 2018 and 2019, no differences were observed in May and July at 0–5 and 5–15 cm depths, while in September and November, drought treatment showed the highest MBC compared to rainfed and irrigated treatments. We observed a general decrease in MBC over time at each sampling depth except the 5–15 cm depth in 2018. Also, there was a significant decrease in MBC with soil depth. Fungal hyphal lengths, measured at 0–5 and 5–15 cm depths, showed similar trends with MBC (Fig. 5a–d). At both depths, fungal hyphal lengths did not show any differences among moisture treatments in May and July, while higher hyphal lengths under drought treatment was observed in September and November compared to rainfed and irrigated treatments in both years. The increased fungal abundance under drought was more evident in 2019 than 2018.

### Extracellular hydrolytic enzyme activity

**Figure 6:**
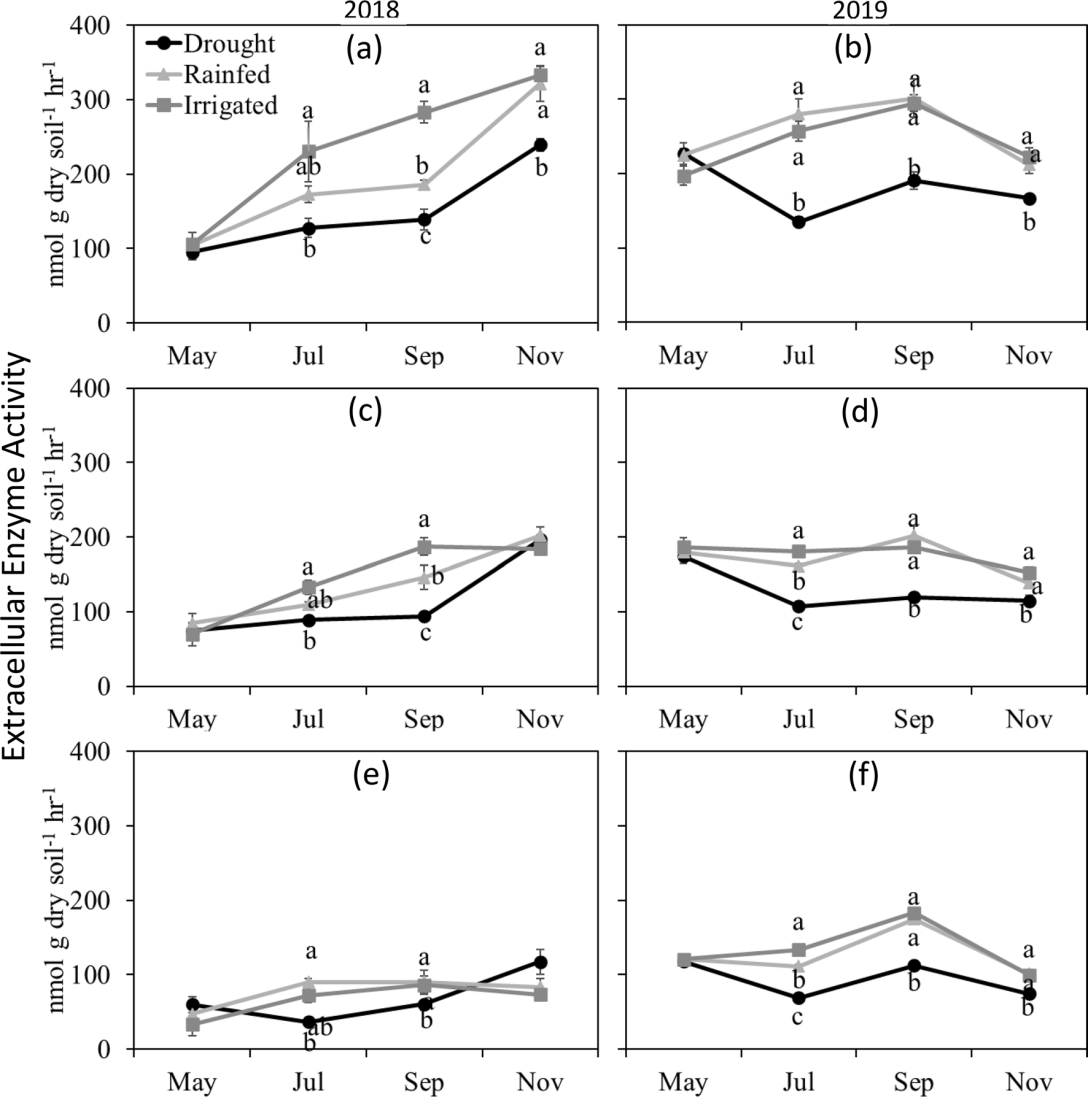
Total extracellular hydrolytic enzyme activity under drought, rainfed, and irrigated treatments at 0–5 (**a**,**b**), 5–15 (**c**,**d**) and 15–30 (**e**,**f**) cm depths during 2018 and 2019. Different letters represent statistical significance at p ≤ 0.05 at each time point. No letters mean no statistical significance. Error bars represent standard error (n = 3). Sampling in May represents baseline and in November represents post shelter removal.

**Figure 7:**
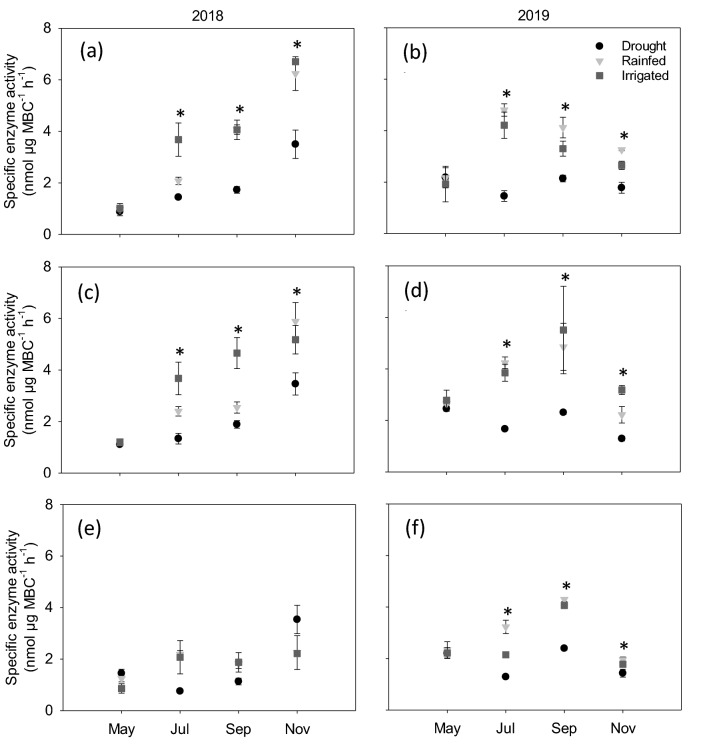
Specific enzyme activity under drought, rainfed, and irrigated treatments at 0–5 (**a**,**b**), 5–15 (**c**,**d**) and 15–30 (**e**,**f**) cm depths during 2018 and 2019. Asterisk represents statistical significance at p ≤ 0.05 at each time point. No asterisks mean no statistical significance. Error bars represent standard error (n = 3). Sampling in May represents baseline and in November represents post shelter removal.

In general, the total activity of six extracellular hydrolytic enzymes increased with increase in soil moisture (Fig. 6). For all depths, drought treatment showed the lowest activity compared to rainfed and irrigated treatments when treatment differences were significant. In both 2018 and 2019, no differences in enzyme activities were observed in May at all depths (Fig. 6a–f). The lowest enzyme activities under drought as compared to the rainfed and irrigated treatments were also observed in November, 6 weeks after the shelter removal (Fig. 6a–d). Also, the enzyme activity decreased with soil depth. Specific enzyme activity, extracellular enzyme activity per unit MBC, was also similar among the moisture treatments in May 2018 and 2019 (Fig. 7). At other time points, drought treatment showed the lowest specific enzyme activity at 0–5 and 5–15 cm depths in 2018 (Fig. 7a,c) and at all the depths in 2019 (Fig. 7b,d,f).

## Discussion

### Soil moisture changes and microbial activity

In accordance with our hypothesis, the lowest CO_2_ efflux was observed under drought treatment compared to rainfed and irrigated treatments (Fig. 3c,d). Several past studies also reported decreased microbial respiration under water limitation^51–54^. Some studies attributed this to reduced physical accession of C substrates by microbes under moisture deficit^55–57^. Although CO_2_ emissions were greater under rainfed and irrigated conditions compared to drought condition, no significant differences were observed between rainfed and irrigated treatments. This could possibly be due to slight difference in total amount of water received by both these treatments during the study period. The rainfall received during both 2018 and 2019 was sufficient for the growth of soybeans, due to which the sensor-based irrigation system applied only a little additional amount of water in the irrigated treatment. Drought condition led to decreased CO_2_ emissions within 2–3 weeks of shelter establishment (Fig. 3c,d), revealing that only a short length of time following drought onset is required to adversely affect the microbial activity. Once the shelters were removed to allow the precipitation to fall in the drought-simulated plots in October, similar soil CO_2_ efflux was observed among moisture treatments. Notably, during this post-shelter removal period, the CO_2_ emissions from all three moisture treatments were reduced (Fig. 3c,d). This could plausibly be explained by lower soil temperatures in November in both the years (7 °C in 2018 and 8 °C in 2019) (Fig. 2). Also, due to crop harvest in October, the rhizosphere activity of soil microbes would have been drastically reduced, leading to lower CO_2_ emissions.

Extracellular enzymes regulate the cycling of soil organic matter and nutrients. Consistent with the soil CO_2_ efflux results, extracellular enzyme activity also decreased under drought (Fig. 6). This could be attributed to diffusion constraints from moisture limitation, limiting interaction of enzymes with their respective substrates^58^ and slowing down the decomposition^59,60^. Also, as soils dry, enzymes may be adsorbed to clay minerals more tightly, making them more resistant to proteolytic breakdown^61^. Under drought conditions, enzyme production and activity are reduced as the nutrient requirement for enzyme production exceeds the net increase in nutrient availability for microbes^62^. Interestingly, even under drought, we observed the maintenance of enzyme pool which could possibly be explained by reduced enzyme turnover due to stabilization under moisture limited conditions^63^ or by continuous supply of substrates in the form of plant inputs for the enzymes to act on^12^. The decrease in enzyme activity with depth was observed, which could be due to the decline in the relative contribution of plant derived C, particularly in our no-till system^64^ or decrease in MBC with depth (Fig. 5).

The reduced enzyme activity in drought treatment continued even after the shelters were removed and all the treatments had received multiple rain events. This indicates that microbes under drought conditions may have acclimated or shifted in composition in response to the moisture deficit conditions and continued to metabolize at lower rates even when the soil moisture was increased, a potential indication of legacy effect of drought^28,29^. This legacy effect of moisture manipulation, however, did not persist between drought cessation in 2018 and onset in 2019 as shown by CO_2_ efflux data, indicating the short-term nature of such effect in this study. The potential legacy effect of drought on microbial activity has been reported by previous studies^28,65–68^. We measured the enzyme activities and CO_2_ respiration for only 6 weeks after the shelter removal, so we were unable to determine how long the legacy effect of moisture deficit lasted. We were also not able to pinpoint the mechanisms of the observed short-term legacy effect as we did not determine microbial community composition or physiological changes Nonetheless, the occurrence, intensity, duration, and mechanisms of legacy effect upon cessation of moisture stress is not studied in detail^25^.

### Soil moisture changes and active C pools

Active soil C fractions have been suggested as more sensitive metrics for detecting the impacts of short-term and extreme changes in environmental factors on total SOC pool^69^. This study considered two active C fractions, EOC and MBC. We observed higher concentrations of EOC under drought treatment compared to rainfed and irrigated treatments (Fig. 4). Several past studies also found increased EOC under water deficit conditions and attributed that to, (1) reduced microbial activity, thus less uptake of EOC^70–73^, (2) sustained enzyme activity under drought treatment even under reduced substrate diffusivity^74,75^, or (3) production of extracellular polymeric substances (EPS)^76^. Additionally, constant plant inputs in the form of root exudates and leaf litter throughout the growing season coupled with lower microbial activity and uptake due to water stress may also have resulted in EOC accumulation in soils under the drought treatment^77^.

The MBC, another active C fraction, also increased under drought condition (Fig. 5). This is different from the general understanding that moisture deficit conditions impose direct physiological stress on microorganisms^78^, eventually leading to decreased microbial biomass. However, in accordance with our findings, several previous studies have also reported increased MBC under drought conditions^72,79–81^. There are evidences that certain microbial taxa are tolerant to drought stress^82^ and continue to grow under stressful conditions^83^, especially fungi^11^. Fungi are considered to be more tolerant to moisture stress due to the accumulation of osmoregulatory solutes^84^ and also due to their filamentous structure that facilitates greater access and utilization of substrates even under drought conditions^85^. We also found increased fungal hyphae abundance under drought conditions (Fig. 5), revealing greater fungal survival under moisture stress in our study. Obviously, there could be other mechanisms for increased MBC under moisture stress that are not determined in this study. For example, microbial death rates may be slower under drier conditions as the movement of microbial predators (e.g., protozoa, nematodes, phage) through soil water is reduced^36^. This decreased microbial death rates under drought conditions could reflect as increased MBC. Accumulation of intracellular osmolytes^86^ or presence of EPS^33,76^ may also contribute to higher MBC in moisture deficit conditions.

### Soil moisture changes and microbial metabolic status

The specific enzyme activity, which is the total extracellular enzyme activity expressed per unit MBC, represents the metabolic status of the soil microbial community^87^. This metric normalizes differences in MBC to provide a reliable comparison of metabolic status of microbes under different soil moisture treatments. Our study yielded the lowest specific enzyme activity under drought treatment compared to rainfed and irrigated treatments (Fig. 7) and that is because the increase in MBC under drought was higher (Fig. 5), than the decrease in total enzyme activity (Fig. 6) relative to rainfed and irrigated treatments. The decreased specific enzyme activity under moisture limitation is also due to immobilization of the extant pool of enzymes by forming complexes with soil particles and becoming unavailable for proteolytic breakdown^88^. Greater specific enzyme activity in rainfed and irrigated treatments indicates more metabolically active microorganisms. More metabolically active microbes undergo enhanced turnover^89^, thus explaining the lower MBC values in these treatments (Fig. 5). Also, active microbes can increase synthesis, production, and release of enzymes, as seen in our study (Fig. 6). This greater specific enzyme activity aligns with higher CO_2_ respiration when moisture was not limiting, which also leads to greater microbial turnover. Even though the total extracellular enzyme activity was increased when moisture was not limiting (Fig. 6), we are unable to deduce if the higher enzyme activity in rainfed and irrigated treatments is due to alleviation of diffusion constraints by increased soil moisture, synthesis of new enzymes, higher microbial activity, or combination of all of these. Taken all these results together, our study revealed that under drought conditions, the active fraction of microbes is lower, despite the higher amount of total microbial biomass.

## Conclusion

This 2-year in situ drought manipulation study showed reduced CO_2_ efflux and enzyme activity when soil moisture was limited compared to rainfed and irrigated conditions that maintained higher soil moisture content. However, total MBC and EOC were increased under drought treatment. Concomitant with the increase in MBC, fungal hyphae abundance was also increased in the drought treatment. The effect of soil moisture changes to microbial C cycling was more evident in the top 15 cm of the no-tilled soil than 15–30 cm, which could be due to that surface soil experiences moisture stress faster than subsoil when drought occurs. In addition, SOC density is typically stratified with depth in the no-tilled soil. We also observed that despite higher MBC, microbes were less metabolically active under drought conditions indicating presence of higher proportion of dormant microbes. Findings from this in situ moisture manipulation study reflect dynamic interactions between soil, plants, microorganisms, and climate. These findings improve understanding of the role of moisture on microbial C cycling and the possible determinants including microbial biomass, enzyme activities, and EOC concentrations that control CO_2_ fluxes from managed ecosystems.
